# Effectiveness of Mindfulness-acceptance-commitment based approach for Rumination, Cognitive Flexibility and Sports Performance of Elite Players of Beach Soccer: A Randomized Controlled Trial with 2-months Follow-up

**DOI:** 10.2174/17450179-v19-e230419-2022-33

**Published:** 2023-05-12

**Authors:** Fatemeh Sabzevari, Hossein Samadi, Farahnaz Ayatizadeh, Sergio Machado

**Affiliations:** 1 Department of Sports Psychology, Yazd University, Yazd, Iran; 2 Department of Physical Education and Sports Science, Faculty of Psychology and Educational Sciences, Yazd University, Yazd, Iran; 3 Department of Sports Methods and Techniques, Federal University of Santa Maria, Santa Maria, Brazil; 4 Laboratory of Physical Activity Neuroscience, Neurodiversity Institute, Queimados- RJ , Brazil

**Keywords:** Mindfulness-commitment-acceptance, Cognitive flexibility, Rumination, Sports performance, Athletes, Cognitive flexibility

## Abstract

**Background/Objective:**

There is little research on the effectiveness of new approaches to psychology, including mindfulness-acceptance-commitment, especially in team disciplines. Therefore, this study compared mindfulness-acceptance and commitment-based approaches to rumination, cognitive flexibility, and sports performance of elite beach soccer players during a two-month follow-up.

**Methods:**

The research design consisted of a randomized controlled trial (RCT), with follow-up. Thus, 34 players of the premier league of beach soccer were randomly divided into intervention and control groups based on mindfulness acceptance and commitment. The experimental group exercises consisted of one session per week for 7 weeks and daily homework. Participants filled out the questionnaires of the Ruminative Response Scale, Cognitive Flexibility Inventory, and Sports Performance Questionnaire before, after, and at two months of follow-up of the intervention.

**Results:**

Multivariate Analysis of Variance (MANOVA) and Analysis of Variance (ANOVA) with repeated measures were used to evaluate the changes over time and compare the scores of the subjects of the two groups. The results showed a significant difference in mindfulness-acceptance and commitment intervention in the experimental group on pre- *vs.* post-test and pre-test *vs.* follow-up scores of research variables. Also, a comparison of groups using independent T-test analysis showed a significant effect of mindfulness-acceptance and commitment exercises on research variables in the experimental group in the post-test and follow-up stages.

**Conclusion:**

Findings suggest that mindfulness, commitment, and acceptance exercises can be used as a new method to reduce rumination and increase cognitive flexibility and sports performance of elite beach soccer players.

## 1 INTRODUCTION

As long as athletes strive to achieve optimal performance, psychological interventions help develop skills and maintain their performance [1]. In other words, the focus of sports psychology is to determine and understand theories and techniques to improve the optimal performance of athletes in the face of stressors to achieve success in sports [2]. Rumination is one of the problems with the content of thoughts affecting the performance level of athletes. A ruminant focuses on negative emotions and feelings, repetitive, interventionist, and negative thoughts [3], which leads to increased stress. In the theory of Nolen-Hooksma response styles, rumination is defined as repetitive thinking about failure symptoms, possible causes, and consequences, which intensifies and perpetuates a person's problems by increasing negative thinking, inefficient problem solving, interfering with purposeful behavior, and decreasing social support [4]. Rumination can be one of the most important problems of athletes, especially professional athletes at different championship levels, and may distract the athlete's attention from important topics and goals [5]. Experimental avoidance and psychological resilience are two dimensions of a continuum, and the closer a person gets to one of them, the closer they move away from the other. Empirical avoidance refers to the avoidance of negative inner experiences, and empirical acceptance refers to experiencing a wide range of emotions [6]. Achieving the psychological ability causes one to accept and face disturbing experiences instead of escaping negative thoughts and emotions and trying to control them. Improving cognitive flexibility leads to functional differences acceptance, reduces cognitive tendencies, and prevents the stimulation of ironic mental processes [7, 8].

In recent years, experimental studies have extensively focused on individual psychological factors, including rumination, cognitive flexibility, and their effects on performance [9, 10]. It seems that engaging in automated processing processes rather than mindfulness-based processes combined with inflexible attention and lack of focus on the present moment causes people to think more about failure and negative self-evaluation, and causes rumination in them [11]. Studies have shown that negative evaluations and excitements during the competition have a disabling effect on the athlete's future performance and affect the athlete's behavior and performance due to a lack of control over thoughts and symptoms [12]. Also, for athletes, a high level of experimental avoidance can be problematic because athletes are usually in a situation where they may suffer from a lot of stress and physical fatigue [13].

In the sports field, applied psychology mainly involves the use of traditional cognitive-behavioral methods and techniques that mainly focus on developing self-control of internal states, which are commonly referred to as Psychological Skills Training (PST) [14, 15]. Indeed, the traditional psychological approach is based on the hypothesis that negative inner states, such as negative thoughts, emotions, and negative physical emotions, prevent optimal functioning. Also, to increase performance, they must be controlled, stopped, or reduced by increasing positive thoughts and self-confidence [1, 16]. Although the role of cognitive-behavioral strategies has been confirmed for controlling emotions, facilitating adaptation to competitive pressure, and reducing negative internal experiences among athletes in various disciplines and in recreational, academic, professional, and olympic athletes, studies have not shown significant improvement in sports performance [1, 17].

Recent studies have challenged the hypothesis that negative internal experiences always lead to negative behavioral consequences despite some positive results of traditional psychological interventions. Studies suggest that trying to suppress negative thoughts and patterns of thought in action can increase unwanted cognitive activity (*e.g.*, increasing the occurrence and repetition of unwanted thoughts and emotions), which ultimately increases self-awareness, thereby leading to impaired performance [1, 15, 17]. According to ironic mental processes, trying to control or suppress a particular thought may increase conflict in the same thought, which one is trying to avoid. In addition, self-focused attention on automatized movements and trying to control them may be associated with decreased sports performance [18]. Indeed, unlike control-based interventions, which consider the optimal internal state necessary for performance [1], today we are faced with the third generation of integrated and new therapies, such as cognitive therapy based on mindfulness and therapy based on acceptance and commitment, which is different from the traditional approach to practicing psychological skills, both in terms of theoretical hypotheses and intervention strategies [17, 19, 20].

Mindfulness Acceptance Commitment (MAC) approach to performance enhancement is one of the newest psychological interventions recently developed for athletes. Indeed, the MAC approach uses acceptance and commitment therapy and adds a mindfulness component to this model that offers a different perspective that challenges traditional interventions commonly used by psychologists [1, 21]. Attention to the present moment is the core of mindfulness and means the ability to change attention to the present, and is defined in a flexible and non-judgmental way in the face of internal and external stimuli by impartial observation [7, 21]. Acceptance is defined as the ability to actively and consciously accept any private event (embrace) without attempting to avoid it or change its intensity or form [22]. Values and committed actions mean recognizing what is important to the people setting their goals, and acting responsibly and effectively to achieve their goals [23, 24]. The goal of the intervention is to promote cognitive flexibility to help people live following personal values and goals. Underlying processes, such as acceptance, cognitive defusion, awareness of the present moment, and value, have significant effects on behavioral changes in a wide range of contexts [25]. This intervention aims not to make a direct change in the clients. Still, its purpose is to help the clients to be able to make a connection with their experiences in different ways. It allows athletes to focus on external movements rather than internal self-referential thoughts, which is a motor learning principle [26]. In the new interventions, including the MAC, value-based behaviors are trained instead of emotionally-oriented behaviors to increase people's commitment to their goals [27].

The MAC helps athletes develop the skills of attention, awareness, judgment, empirical acceptance, and inner experience, while focusing on performance-appropriate behaviors to enhance competitive instant behaviors. This approach also helps athletes identify emerging disruptive emotions, whose lack of control can lead to cognitive and behavioral maladaptive reactions and adversely affect athletic performance [27]. Although the effects of MAC intervention on performance are significant, it is equally critical to discover the underlying mechanisms of these effects on performance. According to Gardner [28], MAC intervention may not have a direct impact on performance; indeed, it indirectly leads to improved performance through another variable [28]. In a study, Josefsson *et al*. [15] examined the effect of the mindfulness-commitment approach on the regulation of specific sensory focus and athletic performance in multiple sports groups. This study was carried out on 69 competitive elite athletes who had no previous experience in mindfulness and acceptance in both MAC and PST groups. The results showed MAC intervention to have an indirect effect on exercise performance and showed a significant improvement in emotion regulation and sports mindfulness compared to the PST group [15]. In a study, Lundgren *et al*. [21] examined the effectiveness of an acceptance and commitment program in improving the cognitive flexibility of 21 players in ice hockey. The 4-week acceptance and commitment program showed a significant increase in the flexibility of the intervention group [21]. Also, Robinson and Cedenblad [29], in a study entitled the effect of MAC intervention on performance and healthy of competitors of martial sports, found MAC intervention as an effective approach to the improvement of performance and health of the subjects and to help develop the ability to pay attention, awareness, and acceptance regardless of thoughts and feelings [29]. In a study on female student-athletes, Gross *et al.* [30] did not observe a significant difference between MAC and PST groups in athletic performance. However, they showed psychological distress and increased psychological flexibility from after the intervention to one month of follow-up [30]. Plemmons [31], in a study, showed the effectiveness of MAC training for the performance of recreational golfers. However, the results showed a significant increase in levels of mindfulness and a tendency to control anxiety in the MAC group compared to the control group. Still, no significant changes in the performance were found after the MAC intervention [31].

Despite the growing popularity of mindfulness, acceptance, and commitment-based interventions in sports psychology, the evidence-based effectiveness and method are limited. The research quality in this area is poor [21]. Also, despite the need to examine the long-term effects of interventions (efficiency), few studies have followed up on their interventions and reported only immediate effects (effectiveness) of their programs [32]. The findings in this field are contradictory. Some previous research has included different samples, but it did not include a control group and sports performance evaluation [33]. Considering the negative effects of rumination and experimental avoidance on athletes' performance and that no similar research has been conducted in this area, this study aimed to compare the effectiveness of mindfulness-acceptance and commitment-based approaches for rumination, cognitive flexibility, and sports performance of elite players of beach soccer.

## 2 MATERIALS AND METHODS

### 2.1 Sample and Procedures

This was a randomized controlled trial (RCT) with a follow-up design. The study was carried out on beach soccer players with at least 6 years of sports experience who were voluntarily invited to participate in the study. The samples were selected using the research consent form, mental and physical health form, and activity history. All athletes had at least six years of regular activity in beach soccer and experience playing for the national team and the Premier League. The specimens were in perfect health physically and psychologically. According to information obtained from coaches and athletes, none of the people had a history of attending psychological classes, and it was their first experience. The written consent and the commitment form to participate in the class and the test were taken from the subjects after selecting the eligible individuals based on the information obtained from the questionnaire and explaining the purpose to the subjects. Forty subjects were randomly divided into mindfulness-acceptance and commitment intervention (n=20) and the control group (n=20). At the end of the intervention, one person from the experimental group and five people from the control group were excluded for not participating in the intervention and completing the questionnaires. The psychological training program was performed under the supervision of a researcher through the assistance of an experienced clinical psychologist. According to the protocol of Gardner and Moore [27], subjects in the experimental group participated in 7 specified training sessions during seven weeks (one session per week for about 60-90 minutes). The content of the program included: 1) preparing athletes for psychological training, 2) introducing mindfulness and cognitive defusion, 3) introducing values-based behaviors and values, 4) introducing acceptance, 5) increasing commitment, 6) balancing and consolidating skills, combining mindfulness, acceptance, and commitment, and 7) maintaining and strengthening mindfulness, acceptance, and commitment, discussion and homework. The control group did not receive any intervention during this period. Data were collected before the first session, after the seventh session, and two months after the last session.

 The purpose and method of conducting the study were explained to all participants in the study. They were given the necessary assurance about the confidentiality of the information and the right to withdraw from the study. Then, written informed consent was obtained from them. This study was approved by the Ethics Committee at Yazd University and was conducted according to the ethical declaration of Helsinki.

### 2.2 Measures

#### 2.2.1 The Ruminative Response Scale (RRS)

The ruminative response scale (RRS) developed by Nolen-Hoeksema and Morrow [3] is a self-test questionnaire that assesses four different styles of negative mood response. The scale measures the ruminant response style, which is negative and inconsistent. The scale has 22 items with four options based on the Likert scale, whose options are scored from the range of never to forever. A score of 33 is the cut-off point of the questionnaire, and scores below 33 indicate low rumination and higher scores indicate high rumination. Yook *et al*. [34] reported an alpha coefficient of 0.90 and a retest validity of 0.68 for this scale.

#### 2.2.2 Cognitive Flexibility Index (CFI)

The Cognitive Flexibility Questionnaire assessed cognitive flexibility. The questionnaire consisted of 20 items and evaluated the cognitive flexibility needed to succeed and replace dysfunctional thoughts with more effective ones. The researchers reported the similarity of the questionnaire as 0.91 and 0.81 in terms of Cronbach's alpha and retest methods, respectively [35].

#### 2.2.3 Charbonneau Sports Performance Questionnaire

To assess sports performance, Charbonneau's Sports Performance Questionnaire was used. The questionnaire has five items based on the Likert scale, and it has been developed for assessing athletes' performance and will be used by the relevant coach of each athlete or by the athlete. The obtained scores show the final score of the athlete's performance. Each question is scored from one (poor) to five (excellent). The scores obtained from the five questions are added together, and the final score of the athlete's performance is obtained. The final score of the athlete's performance is in the range of 5 to 25. The average reliability coefficients of the questionnaire were calculated to be 0.71 by Charbonneau [36].

### 2.3 Statistical Analysis

In this study, data were analyzed by inferential statistics, and descriptive statistics was used to categorize the information and present the mean and standard deviation. Shapiro-Wilk test was used to determine the normality of the data. Also, a MANOVA and an Analysis of Variance (ANOVA) with repeated measures were used to evaluate the changes over time and compare the scores of the subjects of the two groups. In all tests, the confidence level was considered 0.05. Data were analyzed using the SPSS 20 software.

## 3 RESULTS

The demographic information of the research participants is presented in Table **1**.

Results show changes in the scores of cognitive flexibility, rumination scores, and sports performance from pre-test to follow-up in the experimental and control groups, respectively (Fig. **2A**-**C**).

Given the presumptions of the MANOVA statistical test, changes in the scores of cognitive flexibility, ruminative, and sports performance scores in the experimental and control groups were analyzed using MANOVA in the three stages of pre-test, post-test, and follow-up. The results of the test showed significant multivariate effects for the group (Hotelling's Trace=0.49, F_(3,30)_=4.9, P=0.007, η^2^=0.33), time (Hotelling's Trace=4.58, F_(6,27)_=20.61, P<0.001, η^2^=0.82), and the interaction of group and time (Hotelling's Trace=1.92, F_(6,27)_=8.64, P<0.001, η^2^=0.66). The results of the between-group univariate analysis showed the experimental group to have significantly better scores in the variables of cognitive flexibility (F_(1,32)_=4.85, P=0.03, η^2^=0.13), rumination (F_(1,32)_ =4.18, P=0.04, η^2^=0.12), and sports performance (F_(1,32)_=7.62, P=0.009, η^2^=0.19). The results of the within-group univariate analysis indicated the score of cognitive flexibility (F_(1.22,39.11)_=8.18, P=0.04, η^2^=0.20), rumination (F_(2,64)_=4.82, P=0.01, η^2^=0.13) and sports performance (F_(2,64)_=3.38, P=0.04, η^2^=0.10) to be improved significantly over time (regardless of the group factor). There was a significant interaction found between group and time in the scores of cognitive flexibility (F_(1.22,39.11)_=5.17, P=0.02, η^2^=0.14) and sports performance (F_(2,64)_=4.52, P=0.01, η^2^=0.12). However, no significant difference was observed for rumination scores (F_(2,64)_=1.43, P=0.24).

Considering the significance of the interaction of group and time in the variables of cognitive flexibility and sports performance, additional analyses were performed to determine the source of the interaction. In this vein, the within-group comparison of the scores in the studied variables was done using the repeated measures ANOVA and Bonferroni's *post-hoc* test. The results of pairwise comparisons with Bonferroni correction regarding the cognitive flexibility variable showed a significant difference between the pre-test and post-test and between pre-test and follow-up in the experimental group (P<0.01); however, there was no significant difference found between the pre-test and follow-up in the experimental group as well as between different stages in the control group (P>0.05). The results of pairwise comparisons with Bonferroni correction regarding sport performance showed a significant difference between the pre-test and post-test stages and between the pre-test and follow-up stages regarding the sports performance variable (P<0.05); however, there was no significant difference found between post-test and follow-up in the experimental group as well as between different stages in the control group (P>0.05).

Considering the significance of the main effect of time on the rumination variable, the repeated measures ANOVA was used to investigate the place of the differences in order to compare the within-group scores between the stages. The results showed a significant difference between the stages in the experimental group (P=0.05). The results of the pairwise comparisons with Bonferroni correction indicated a significant difference between the pre-test and post-test scores. However, there was no significant difference found between the pre-test and follow-up and post-test and follow-up scores. Also, the results of the repeated measures ANOVA showed no significant difference between the different stages in the control group (P>0.05). Given the significance of the interaction of group and time, independent samples *t*-test was used to compare the scores of two groups in different stages. The results are presented in Table **2**.

## 4 DISCUSSION

This is the first study to investigate the effectiveness of mindfulness, acceptance, and commitment-based approach for rumination, cognitive flexibility, and sports performance of elite beach soccer players in a two-month follow-up. Findings showed a significant difference in MAC intervention effect on the experimental group's pre-test-post-test and pre-test-follow-up scores of cognitive flexibility and sports performance. However, there was no significant difference observed in the post-test-follow-up stages. The results regarding rumination showed a significant difference between the pre-test and post-test stages. Also, a comparison of groups using independent T-test analysis showed the significant effect of mindfulness-acceptance and commitment exercises on research variables in the post-test and follow-up stages in the experimental group.

The results of the flexibility variable showed that in the experimental group, unlike the control group, cognitive flexibility scores after seven weeks of a mindfulness intervention, commitment acceptance in the post-test phase increased significantly, and this effect was maintained until the follow-up phase. Unlike the traditional approach, the MAC approach emphasizes that there is no need to remove, change, or control emotional and inner cognitive levels to improve performance; rather, the development of mindful thinking, the acceptance of present inner experiences (such as thoughts, emotions, and bodily sensations), the clarification of valuable goals, and the increasing attention to external cues, responses, and possibilities should be considered. Rather, the development of mindful thinking, the acceptance of present inner experiences (such as thoughts, emotions, and bodily sensations), the clarification of valuable goals, and the increasing attention to external signs, responses, and probabilities should be considered. The MAC approach combines four approaches of attention awareness, acceptance, commitment, and behavior change, and its overall goal is ultimately to achieve psychological flexibility, so that there is no need to eliminate the bad feeling. Rather, despite this feeling, one moves towards thought-based behavior to increase one's psychological connection with thoughts and feelings instead of changing cognitions [1, 27].

In mindfulness-based therapy, acceptance and commitment are believed to produce a natural mind. What turns thoughts into belief is incorporating the individual into the content of the thoughts [27]. In this type of intervention, the athlete is taught to observe their thoughts, emotions, and feelings without judgment and see them as simply mental events, instead of seeing them as part of themselves or a reflection of reality [37]. It seems that MAC improves attention span and awareness of current thoughts and feelings, which may help people quickly identify annoying emotions that need to be adjusted [38]. Increasing emotional awareness after MAC intervention helps athletes quickly recognize emerging destructive emotions, whose lack of control can lead to cognitive and behavioral maladaptive reactions and adversely affect sports performance. Increasing the ability to accept emotions and emotional states prevents athletes from performance-inhibiting emotions. In addition, the MAC approach helps athletes reduce experiential avoidance and increase their tolerance for unpleasant situations; so, the processes can make it easier for athletes to pursue value-based behaviors *versus* emotion-centered avoidance behaviors and increases individual commitment and adherence to goals [27, 38]. One of the most important techniques in explaining the above hypothesis is the MAC approach, specifying values and committed actions. In this way, people learn not to consider life and activities as goals or results but as a process or a path. This approach emphasizes accepting unchangeable something as a tool for recognizing and changing changeable things. Focusing at the present moment prevents one from delving into judgments about one's past and future unpleasant experiences. The process of contact with the moment of life helps people make the situation more bearable and accept the experience of real-life moments that make cognitive flexibility.

The results also showed mindfulness-commitment acceptance exercises to affect the rumination of elite players of beach soccer and reduce the rumination of players in the post-test stage. Ruminants create a defective cycle in the thought process that is inefficient and harmful and prevents adaptive problem-solving. Therefore, this cycle must be broken, and people’s psychological flexibility must be increased to behave in the best possible way in different situations [39, 40]. The basis of the acceptance-commitment approach is that if a person is faced with unavoidable situations that are beyond his/her control, the constant mental struggle with those situations only exacerbates his/her feelings of exhaustion and helplessness. While accepting the situation can lead to peace of mind and reduce unpleasant emotions, such as anxiety and rumination. The central process of acceptance and commitment-based approach also teaches people how to stop rumination thoughts and not focus on disturbing thoughts, an issue that makes a person more tolerant of negative emotions [23]. Also, in this approach, the conscious attention of the mind to the present moment and the ability to control and pay more attention to prevent rumination are very effective [41, 42].

The MAC approach increases overall acceptance and leads to the conscious acceptance of disturbing thoughts and feelings. Suppose athletes can accept themselves as they are. In that case, it may be easier for them to accept unpleasant thoughts and feelings, as well as unexpected and unwanted events during training and competition. Increasing the acceptance of mistakes made during competitions can make it easier for athletes to let go of their judgments and thoughts about negative emotions [43].

In general, in this approach, value-based behaviors are developed instead of emotion-oriented behaviors to promote people's constant commitment to their goals [27]; also, it encourages people to accept thought processes as a necessary and real function of psychological adjustment, which in turn reduces negative cognitive schemas [44]. Acceptance and commitment intervention aims to train athletes to change how the athlete relates to private experiences [45]. This approach also uses various techniques to help athletes and practitioners accept their inner selves. At the same time, they focus on stimuli and related tasks to achieve their meaningful values and goals. The focused center allows learners to focus on external movements rather than their internal thoughts. Value-based behaviors are also developed instead of emotion-driven behaviors to enhance individuals' constant commitment to goals [27]. It encourages these people to accept thought processes as a necessary function for psychological compatibility, which reduces negative cognitive schemas [44].

The results showed mindfulness-commitment training exercises to affect the performance of elite players in beach soccer and improve sports performance in the post-test and follow-up stages. The MAC approach is a combination of acceptance and awareness attention along with commitment and behavior change. Mindfulness strategies reduce exercise-related and unrelated concerns associated with improved sports performance [46]. Improving the skills of present-moment awareness may be crucial to achieving a deeper insight into the cognitive and emotional behavioral patterns through which an athlete can apply acceptance skills [47]. A combination of attention, awareness, and acceptance leads to an overall improvement in adaptive behaviors that are important for improving the performance of athletes who are in challenging and stressful situations [15, 48]. In addition, the acceptance of experience leads to a lack of reactions and equips athletes to meet their challenges [16].

Accepting the trained thoughts and feelings in the MAC method is optimal to use cognitive capacity because the athlete makes no conscious effort to suppress the disturbing mental and emotional content [49]. Without using acceptance skills, focus on the work may be distracted by negative thoughts and disturbing emotions, leading to decreased performance. Inversely, attempts to suppress unwanted thoughts increase the frequency of thoughts that one wants to avoid. The tendency to experience and accept worries reduces the frequency of negative sports thoughts [50]. In the MAC approach, athletes should focus on current task-related stimuli instead of changing or reducing thoughts and accepting any internal stimuli, such as negative emotions, that appear in the present moment [15]. The acceptance and commitment approach allows athletes to focus on external movements over internal self-referential thoughts. Indeed, mindfulness-based interventions, with an emphasis on conscious attention to the present, increase awareness of external, internal, and motor symptoms during exercise. Such interventions can increase the precise processing of new information, providing optimal conditions for adaptive behavioral responses and improving sports performance [47, 51]. Marks [52] states that the athlete may automatically focus on recognizing and directing attention to related stimuli using continuous attention and experimental acceptance through training without neglecting other external information related to the senses or internal information from within the body [52]. Paying attention to external stimuli and increasing attention capability can lead to optimal processing of information, efficient specifying of cognitive references, and lower levels of general activation (obvious effort), thereby increasing athlete's capability to make decision and use defensive and invasion opportunities in the challenging situation [1, 53]. This increase in capacity can be helpful in team sports, such as beach soccer, where rapid changes can lead to scoring opportunities. In addition, increasing self-awareness and accepting mental and emotional content may help focus more on the task, promote value-based behaviors, and reduce avoidance behaviors [53].

The results are in accordance with the findings of Gross *et al*. [30], Josefsson *et al*. [15], and Lundgren *et al*. [21]. At the same time, the results are inconsistent with the findings of Plemmons *et al*. [31]. There may be different reasons for this inconsistency, including differences in the nature of the intervention and the type of program, the location and duration of the program, the skill level of athletes, *etc.* [54].

Among the limitations of this research are sampling in an age group and a specific field. In addition, the small number of research samples is one of the other limitations that should be considered in future research. Future research should examine a wide range of athletes to ensure the effectiveness and efficiency of these programs. Considering the effect of some factors, such as team and individual sports, level of experience, skills, sports history, *etc.*, on the interventions, these factors should be carefully considered in future research. Another limitation of the research was using a program for all group members. Some researchers believe that separate individuals should be selected based on certain scales and the program should be designed individually in order to evaluate the program more accurately [55].

Considering that this study was one of the few studies on the effectiveness of MAC intervention for flexibility, rumination, and athletic performance, it is suggested to design and consider new and effective interventions related to stress and their efficiency in other sports in future research. Comparing the effectiveness of this approach in people with different skill levels is another issue that should be considered. In addition, it is necessary to conduct studies comparing the effectiveness of MAC with the effectiveness of other behavioral-cognitive interventions or brain stimulation techniques.

## CONCLUSION

The existence of unknown psychological aspects in some areas, especially anxiety and sports performance, has led physical education coaches and professionals to work harder to plan accurately for their athletes in various sports. Researchers hypothesize that MAC intervention affects key processes, such as acceptance, mindfulness, and values, and helps athletes shift their attention to related sports tasks in the face of internal states, such as anxiety, frustration, and rumination. This intervention can, thus, be used as a way to improve sports performance. In this study, MAC intervention resulted in increased flexibility and reduced rumination, which may be important in increasing performance. However, more research is needed to prove it.

## Figures and Tables

**Fig. (1):**
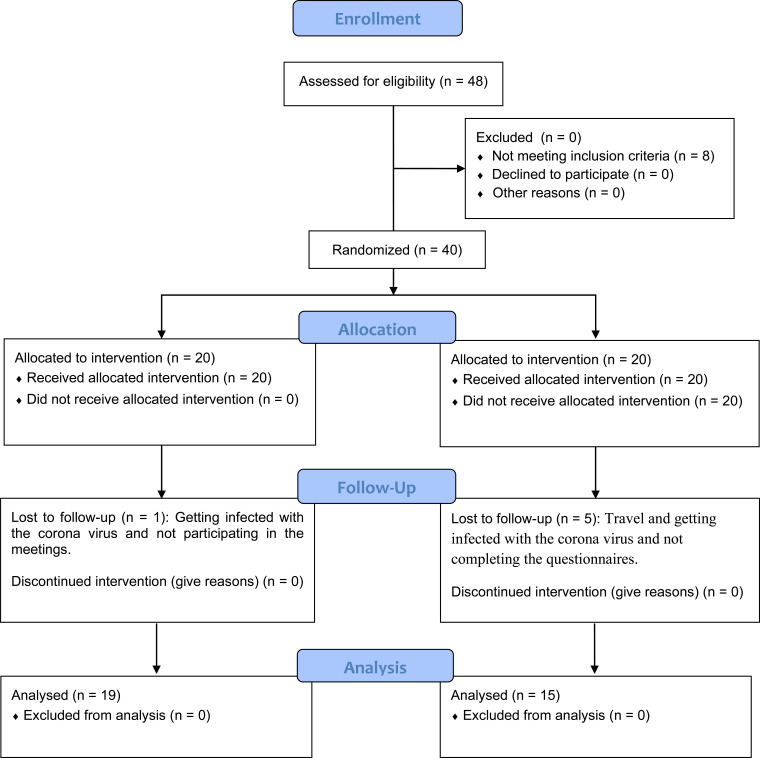
Flow diagram with the experimental phases of the present study.

**Fig. (2A):**
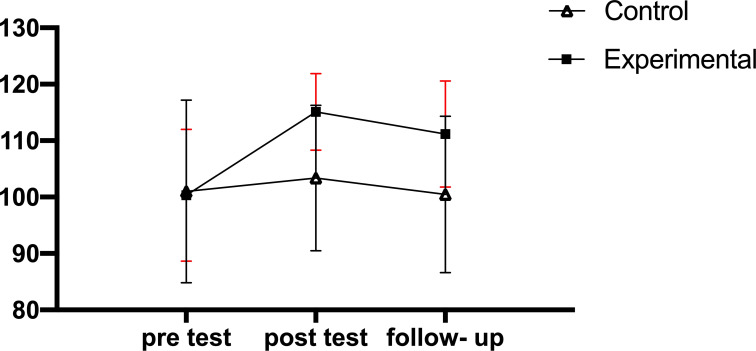
Changes in the cognitive flexibility scores from pre-test to follow-up in the experimental and control groups.

**Fig. (2B):**
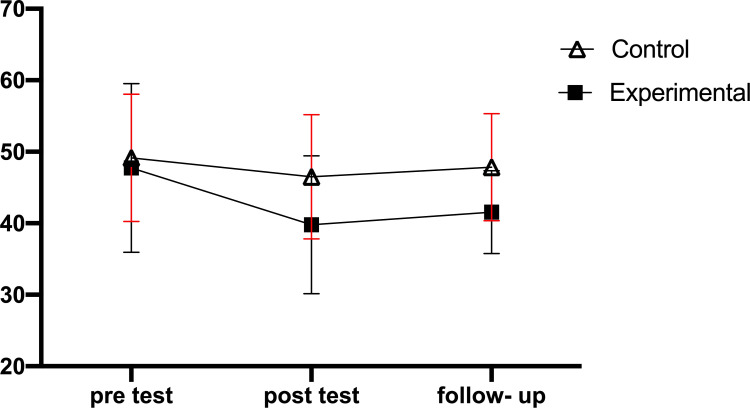
Changes in the rumination scores from pre-test to follow-up in the experimental and control groups.

**Fig. (2C):**
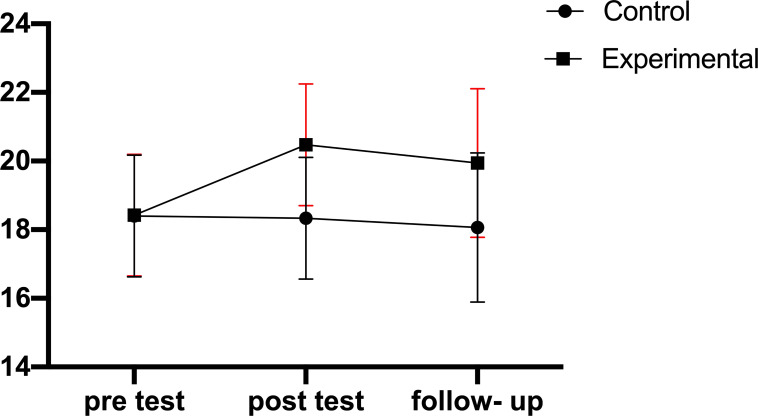
Changes in the sports performance scores from pre-test to follow-up in the experimental and control groups.

**Table 1:** Demographic characteristics of the participants of the five groups.

M ** ± ** SD		Sample	Group Variable
Sports history (y)	Age (y)
6.42 ± 2.52	25 ± 3.62	19	Experimental
7.60 ± 2.66	25.66 ± 5.53	15	Control

**Legend:** M: mean; SD: standard deviation; y: years.

**Table 2:** Results of independent samples *t*-test to compare the scores of two groups in different stages.

** Indicator **	** Stage **	** Group **	** M ± SD **	** T value **	** Degree of Freedom **	** Level of Significance **
** Cognitive Flexibility **	Pretest	Experimental	11.70 ± 3.43	3.40	32	0.002*
		Control				
	Follow-up	Experimental	1.3 ± 40.66	2.67	32	0.012*
		Control				
** Rumination **	Pretest	Experimental	6.73 ± 3.20	2.11	32	0.04*
		Control				
	Follow-up	Experimental	6.2 ± 30.27	2.76	32	0.009*
		Control				
** Sports Performance **	Pretest	Experimental	2.14 ± 0.55	3.82	32	0.001*
		Control				
	Follow-up	Experimental	1.0 ± 88.70	2.68	32	0.01*

**Legend:** M: mean; SD: standard deviation; *significance level at P<0.05.

## Data Availability

All the data and supportive information are provided within the article.

## LIST OF ABBREVIATIONS

RCT: = Randomized Controlled Trial
MANOVA: = Multivariate Analysis of Variance
ANOVA: = Analysis of Variance
RRS: = Ruminative Response Scale
CFI: = Cognitive Flexibility Index
